# FuZhengHuaYuJiangZhuTongLuoFang Prescription Modulates Gut Microbiota and Gut-Derived Metabolites in UUO Rats

**DOI:** 10.3389/fcimb.2022.837205

**Published:** 2022-05-20

**Authors:** Ziwei Chen, Shaobo Wu, Yu Zeng, Zejun Chen, Xueying Li, Jing Li, Long He, Ming Chen

**Affiliations:** ^1^ Department of Nephrology, Affiliated Integrated Traditional Chinese Medicine (TCM) and Western Medicine Hospital of Chengdu University of Traditional Chinese Medicine, Chengdu Integrated Traditional Chinese Medicine (TCM) and Western Medicine Hospital, Chengdu First People’s Hospital, Chengdu, China; ^2^ Department of Nephrology, Hospital of Chengdu University of Traditional Chinese Medicine, Chengdu, China; ^3^ Department of Clinical Laboratory, Hospital of Chengdu University of Traditional Chinese Medicine, Chengdu, China

**Keywords:** FZHY, chronic kidney disease, candidate bacteria biomarkers, gut-derived harmful metabolites, inflammation

## Abstract

**Background:**

Alteration of intestinal flora and metabolites is closely related to chronic kidney disease (CKD) across early to advanced stages. FuZhengHuaYuJiangZhuTongLuoFang prescription (FZHY) is a Chinese herb that has been proven to effectively treat CKD, but the underlying mechanism is not clear.

**Methods:**

Rats were subjected to intragastric treatment with FZHY 7, 14, and 21 days after unilateral ureteral obstruction (UUO) surgery, and kidney tissue, colon tissue, serum, and stool samples were collected.

**Results:**

FZHY treatment effectively ameliorated UUO-induced renal function loss, renal injury and renal fibrosis, and colon tissue damage and fibrosis on day 7. The results of 16S flora analysis (day 7) showed that, compared with the UUO group, both the FZHY group and the sham group showed decreased levels of *g_Monoglobus*, *g_Papillibacter*, *g_Eubacterium_nodatum*, and *g_Family_XIII_AD3011*. Additionally, FZHY obviously induced the reduction of serum citrulline, glycoursodeoxycholic acid, 23-nordeoxycholic acid, 7-ketodeoxycholic acid, kahweol, lipoid B4, 4-(3,4-dihydro-2H-1,5-benzodioxepin-7-yl)-2-methyl-1,3-thiazole, taurolithocholic acid sodium salt, indoline-2-carboxylic acid, 5(S),15(S)-diHETE, and others and the increase of bilirubin, asparagine, and others, which were positively associated with the above four candidate bacteria. Moreover, FZHY increased the levels of ZO-1, occludin, and claudin-1 in the colonic mucosa and reduced the levels of CRP, TNF-α, IL-6, and IL-1 in the serum and LN, FN, Col-I, and Col-III in the tubulointerstitium of UUO rats on day 7.

**Conclusion:**

Our study revealed that FZHY reduced kidney damage at the early stage of CKD by regulating the above four candidate bacteria biomarkers and gut-derived harmful metabolites, inhibiting the inflammation response and tubulointerstitial fibrosis, providing deep insight into CKD therapeutic strategy.

## Introduction

Chronic kidney disease (CKD) is one of the serious public health problems with high incidence, prevalence, and complication (Hasan et al., 2018). Patients with CKD have few signs or symptoms in the early stage of onset, but it mainly manifests as irreversible damage or loss of kidney function until the later stage (Wouters et al., 2015). Pathological changes in the kidneys and other organs caused by CKD generally increase the risk of end-stage renal disease (ESRD) and cardiovascular disease (CVD) and increase mortality (Eckardt et al., 2013). Presently, the main treatments for end-stage renal disease or chronic renal failure are dialysis and kidney transplantation, but the persistently high mortality rate confirms the high socioeconomic burden of the disease. Therefore, there is an urgent need to develop low-cost, high-efficient, and early intervention therapies for CKD.

The gut microbiome is closely linked to the host’s vital organ systems, such as the brain, liver, immune system, and nervous system, and the communication maintains the homeostasis and health of the host (Dinan and Cryan, 2017; Ma et al., 2018; Gomaa, 2020; Morais et al., 2021). However, once this balance is broken, irreversible damage can be done to the whole system. In recent years, there is increasing evidence that the composition and function of intestinal flora have changed and played an important role in the early onset of CKD (Barrios et al., 2015; Yang et al., 2018). The main manifestation is that the increase in urea concentration causes the imbalance of intestinal flora, which in turn mediates the production of intestinal toxins and the destruction of the intestinal epithelial barrier, accelerating the process of kidney injury (Lau et al., 2018; Chen et al., 2019; Hobby et al., 2019). Meanwhile, this process also promotes a systemic inflammatory response, which plays a central role in the pathogenesis of CKD (Andersen et al., 2017). For instance, it is reported that the relative abundance of three species, namely, *Eggerthella lenta*, *Fusobacterium nucleatum*, and *Bifidobacterium animalis*, in a CKD rat model can regulate the toxin output and renal disease development, highlighting the key influence of the gut microbiome on CKD (Wang et al., 2020).

Currently, metabolites derived from intestinal flora are considered as the prerequisite for the progression of CKD, including secondary bile acids, indole, p-cresol (PCS), short-chain fatty acids, lipids, and amino acids (Miyazaki-Anzai et al., 2014; Pickard et al., 2017; Chen et al., 2019; Wang et al., 2020). Indeed, modifying the composition of these metabolites has been found to reduce disease progression in patients and animal models with CKD.

A Chinese medicine prescription is usually composed of a variety of Chinese medicines that play a synergistic role in treating diseases (Yue et al., 2017). Studies have confirmed that Chinese herbal medicine has good pharmacological effects and has been paid more and more attention in the treatment of CKD (Wang et al., 2016; Ma and He, 2018; Chen et al., 2019; Zhao et al., 2020). FuZhengHuaYuJiangZhuTongLuoFang prescription (FZHY) is a traditional Chinese herb composed of nine traditional Chinese medicines, namely, raw astragalus, *Rehmannia glutinosa*, *Salvia*, safflower, wine leeches, soil beetle, wine *Scutellaria*, wine rhubarb, and raw licorice (Chen et al., 2021). The homeostasis of the gut microbiota is closely related to CKD progression, and FZHY has been shown to be effective in the treatment of CKD. Thus, the mechanism of action between FZHY and intestinal flora needs to be further explored. Here, in the study, we established unilateral ureteral obstruction (UUO)-pretreated rats as CKD models and explored the interrelationship among the intestinal flora, serum metabolites, and FZHY to mediate the mechanism of renal damage in rats with CKD, contributing toward understanding the main mechanism of FZHY’s protection of CKD progression, so as to achieve a better therapeutic effect.

## Materials and Methods

### Animals and the UUO Model

Male Sprague–Dawley (SD) rats (7 to 8 weeks, 240–280 g body weight) were obtained from the Laboratory Animal Business Department, Shanghai Institute of Planned Parenthood Research (Shanghai, China). All rats were randomly divided into four groups: sham, UUO, UUO+FZHY, and UUO+AST-120 (*n* = 12 for each group). As described previously (Liu et al., 2017), UUO treatment was used as the CKD disease model (Eddy et al., 2012). The UUO model treatment was as follows: shortly, all rats were anesthetized by intraperitoneal injection of sodium pentobarbital and were fed adaptively for 1 week. The abdominal cavity was opened through the left abdominal incision. The left ureter was bluntly separated and double-ligated with 4-0 sutures in the middle and upper one-third, and the abdominal cavity was closed by layered suture. The rats underwent a similar operation but the ureter was not ligated. The rats were used in accordance with the National Institutes of Health Guidelines for the Use of Laboratory Animals approved by the Ethics Committee of Chengdu University of Traditional Chinese Medicine. FZHY is a Chinese medicine that we developed, and AST-120 has been shown to improve the progression of CKD as a positive control (Schulman et al., 2014). The dose of FZHY for each rat was 4.92 g/kg/day. The dose of AST-120 for each rat was 4 g/kg/day (El-Kafoury et al., 2019). Rats were given intragastric administration once a day for 7, 14, and 21 days, respectively. Then, seven rats were randomly selected from each group and sacrificed. Feces, blood, serum, colon tissue, and kidney tissue were collected for subsequent experiments.

### Histopathological Assay

The histological morphology was evaluated by hematoxylin and eosin (H&E) and Masson’s trichrome staining. The kidney tissues were fixed in 4% paraformaldehyde, embedded in paraffin, and sliced into 4-μm paraffin sections. Then, sections were treated in xylene, dehydrated with graded ethanol, and stained with H&E and Masson (Sigma-Aldrich; Merck KGaA, Darmstadt, Germany). After staining, the sections were dehydrated with 70% and 90% ethanol. Six fields (×200) were randomly selected and observed with an optical microscope (Olympus, Tokyo, Japan). As previously described (Chen et al., 2019), kidney tissue damage such as tubular dilation or atrophy, tubular epithelial cell shedding, and inflammatory infiltration was assessed and scored by three pathologists. The scoring system was as follows: 0 = normal, 1 = mild (<25%), 2 = moderate (26%–50%), 3 = severe (51%–75%), and 4 = extensive (>76%). For the colonic tissue staining, a 5-μm paraffin section was sliced, and the remaining steps were the same as the staining experiments in the kidney tissue. The colonic damage was assessed as previously described with minor modifications such as epithelial cell destruction, goblet cell reduction, and inflammatory cell infiltration (Zheng et al., 2019), also scored by three pathologists. The scoring system was as follows: the epithelial cell destruction, 0 = none, 1 = mild, 2 = moderate, 3 = severe; the goblet cell reduction, 0 = none, 1 = mild, 2 = moderate, 3 = severe; and inflammatory infiltration, 0 = none, 1 = mild, 2 = moderate, 3 = severe. In addition, tubulointerstitial collagen deposition, that is, the blue-stained area, is semiquantitatively calculated as fibrotic areas.

### Immunohistochemistry Analysis

For the immunohistochemistry (IHC) experiments, as above described, 4-µm-thick sections were prepared. The kidney tissues were incubated with antibodies against laminin (LN, CY6617, 1:100, Abways, Shanghai, China), fibronectin (FN, CY5621, 1:100, Abways), collagen I (Col-I, AF0127, 1:200, Affinity, Changzhou, China), and collagen III (Col-III, AF0136, 1:100, Affinity) overnight at 4°C. The colon tissues were incubated with antibodies against ZO-1 (AF5145, 1:100, Affinity), occludin (CY5997, Abways), and claudin-1 (AF0127, 1:200, Affinity) overnight at 4°C. Then, the tissues were incubated with an anti-rabbit secondary antibody (cat. no. ab150077; Abcam, Cambridge, USA) for 2 h at room temperature. Finally, a microscope (Olympus, Tokyo, Japan) was used to capture a representative area containing immunostained tissue (magnification, ×400). The staining intensity score was defined as follows: 0 = negative, 1 = weak, 2 = moderate, and 3 = strong. The positive cell score was defined as follows: 0 = <5%, 1 = 5%–25%, 2 = 26%–50%, and 3 = >75%.

### Real-Time Quantitative Polymerase Chain Reaction Assay

The expression levels of intestinal epithelial adhesion molecules (ZO-1, occludin, and claudin-1) and fibrosis-related molecules (LN, FN, Col-I, Col-III) were detected by real-time quantitative polymerase chain reaction. The total RNA was extracted from intestinal tissue samples using TRIzol reagent from GenePharma (Shanghai, China). A 500-ng RNA was used to synthesize cDNA through Bestar™ qPCR RT kit (DBI Bioscience, Shanghai, China) and then 100 ng of cDNA was used for RT-qPCR using Bestar^®^ SybrGreen qPCR Master Mix (DBI Bioscience, China) according to the manufacturer’s protocol. The reaction consists of the following four steps: 95°C for 5 min, 95°C for 30 cycles, 60°C for 30 s, 72°C for 30 s, and finally 72°C for 7 min. The primer sequences were as follows: ZO-1, 5′-TCGAGGTCTTCGTAGCTCCA-3′ (forward), 5′-GCAACATCAGCAATCGGTCC-3′ (reverse); occludin, 5′-GTGGCTTCCACACTTGCTTG-3′ (forward), 5′-TGTACCCTCCGTAGCCGTAA-3′ (reverse); claudin-1, 5′-TGGGTTTCATCCTGGCTTCG-3’ (forward), 5′-AGCAGTCACGATGTTGTCCC-3′ (reverse); LN, 5′-AATTCCAGGGTGAATGGACGG-3′ (forward), 5′-GTGCACTCCAGTCTTCTGTGG-3′ (reverse); FN, 5′-GATGAGCTTCCCCAACTGGT-3′ (forward), 5′-CTGGGTTGTTGGTGGGATGT-3′ (reverse); Col-I, 5′-GGAGAGAGCATGACCGATGG-3′ (forward), 5′-GGGACTTCTTGAGGTTGCCA-3′ (reverse); Col-III, 5′-GCTCGGAATTGCAGAGACCT-3′ (forward), 5′-AGCATCCATCTTGCAGCCTT-3′ (reverse); and GAPDH, 5′-GAAGGTCGGTGTGAACGGAT-3′ (forward), 5′-ACCAGCTTCCCATTCTCAGC-3′ (reverse). GADPH was considered as an internal control for mRNA. The relative expression of these samples is calculated through the 2^−ΔΔCt^ method, and GAPDH was used to normalize the gene expression.

### Western Blotting

Total proteins were extracted from colon tissue or kidney tissue samples when treated with radioimmunoprecipitation assay (RIPA) lysis (Sangon Biotech, Shanghai, China). The concentrations of total proteins were measured by bicinchoninic acid (BCA) assay (Beyotime, Shanghai, China). The proteins were separated on SDS–polyacrylamide gel electrophoresis and transferred to an NC membrane. The NC membrane was blocked with 5% non-fat milk and then incubated with primary antibodies against ZO-1 (AF5145, 1:500, Affinity), occludin (CY5997, Abways), claudin-1 (AF0127, 1:1,000, Affinity), Col-I (AF0127, 1:2,000, Affinity), Col-III (AF0136, 1:1,000, Affinity), FN (CY5621, 1:1,000, Abways), and LN (CY6617, 1:1,000, Abways) overnight at 4°C, respectively. Then, the membrane was washed with TBST and incubated with secondary goat anti-rabbit (S0001, 1:5,000, Affinity) and goat anti-rat antibodies (S0009, 1:5,000, Affinity) at 37°C for 2 h (Cell Signaling Technology, CA, USA). Finally, the proteins were monitored with enhanced chemiluminescence reagents (GE Healthcare Life Sciences, NJ, USA) and then quantitatively analyzed by ImageJ software (version 1.4.0., National Institutes of Health). GAPDH protein was used for the internal control.

### Enzyme-Linked Immunosorbent Assay

Blood samples from rats were used to detect biochemical markers and cytokine secretion levels. Serum samples were collected by centrifugation at 3,000 rpm for 20 min, and the serum creatinine (SCR, ml058879) and blood urea nitrogen (BUN, ml730662) were measured by ELISA kits (Shanghai Enzyme-linked Biotechnology Co., Ltd., Shanghai, China), respectively. The CRP (ml038253), TNF-α (ml002859), IL-6 (ml102828), and IL-1 (ml037373) were measured by ELISA kits.

### DNA Extraction and 16S rRNA Sequencing

The genomic DNA was obtained from rat feces and extracted using the SDS method, and then, the concentrations and purity of the DNA were detected using a multimode microplate reader (BioTek Instruments, Inc., Winooski, VT, USA). The genomic DNA was used as a template to amplify the V4–V5 region of the 16S rRNA gene using 16s-F and (5′-GTGCCAGCMGCCGCGG-3′) and 16S-R (5′-CCGTCAATTMTTTRAGTTT-3′) primers. Then, a 20-μl PCR reaction program was carried out by adding the Phusion^®^ High-Fidelity PCR Master Mix (Thermo Fisher Scientific, Sunnyvale, CA, USA) with GC Buffer (TaKaRa, Kyoto, Japan) and high-fidelity enzyme. The target bands were recovered using the GeneJET Gel Extraction Kit (Thermo Scientific). Finally, the library was constructed using the TruSeq^®^ DNA PCR-Free Sample Preparation Kit (Illumina, San Diego, CA, USA), and 16S rRNA sequencing was performed on a NovaSeq 6000 (Novogene Bioinformatics Technology, Beijing, China).

### 16S rRNA Analysis

Raw data for 16S rDNA amplicon sequencing were obtained from the NovaSeq 6000 platform in paired-end 250 bases (PE250) mode. Clean data were obtained by Trimmomatic (version 0.36) for data quality control. The operational taxonomic units (OTUs) for clean data were analyzed by Uparse (version 7.0.1001) with the cutoff of 97% sequence similarity. Species annotation analysis was performed using the Mothur method and SSU rRNA database (Wang et al., 2007) of SILVA 138 (http://www.arb-silva.de/). Multiple sequence alignment was performed using the MUSCLE (Edgar, 2004) (version 3.8.31, http://www.drive5.com/muscle/) software to obtain the phylogenetic relationship of all OTU representative sequences. The Shannon and Simpson indices were calculated by using the Qiime software (version 1.9.1), and the outlier index of each group of samples was detected (an outlier is defined as less than Q1-1.5iQR or greater than Q3+1.5iQR, and the outlier is filled with the median of each group of samples). Wilcoxon rank-sum test was used to test the difference of the Shannon and Simpson indices between the two groups. The vegan package of R software (version 3.6.0) was used for PCoA analysis. The significant differences in genus abundance were detected by *t*-test analysis. Functional annotations were performed using Phylogenetic Investigation of Communities by Reconstruction of Unobserved States (PICRUSt) software.

### Metabolomics

Plasma metabolites were used for untargeted metabolomic liquid chromatography-mass spectrometry (LC-MS) analysis (Dunn et al., 2011). The spectrogram processing and database search were carried out by Compound Discoverer 3.1 to obtain the qualitative and quantitative results of metabolites. The logarithmic conversion and standardization of data were carried out by the metaX (Wen et al., 2017) software. Differential metabolites were screened by partial least squares discrimination analysis (PLS-DA) and the threshold was set as VIP >1.0, FoldChange >1.5 or FoldChange <0.667, and *P <*0.05 (Heischmann et al., 2016). Differential metabolites were presented with volcano plots. The Pearson correlation coefficient was used to calculate the correlation between genus abundance and metabolites in R software (version 3.6.1), and the cutoff value (|*R*| > 0.4, *P* < 0.05) was considered a significant correlation. The correlation network is constructed: the edges represent the degree of correlation between the two nodes, and the nodes represent genus or metabolites. Then, the Cytoscape software (version 3.6.1) was used for the visualization and analysis of the correlation network between genus abundance and metabolites, and all the metabolites were classified.

### Statistics Analysis

Statistical data were analyzed using GraphPad Software V8.0 and presented as mean ± standard deviation (SD). One-way or two-way ANOVA (followed by Tukey’s test) was used for difference testing among three groups or more than three groups. Each experiment was performed on three separate assays. *P <*0.05 was considered a significant difference, and *P <*0.05, 0.01 were marked as *, **; #, ##; and Δ, ΔΔ.

## Results

### FZHY Treatment Improved Kidney Function and Morphological Changes in UUO Rats

**Figure 1:**
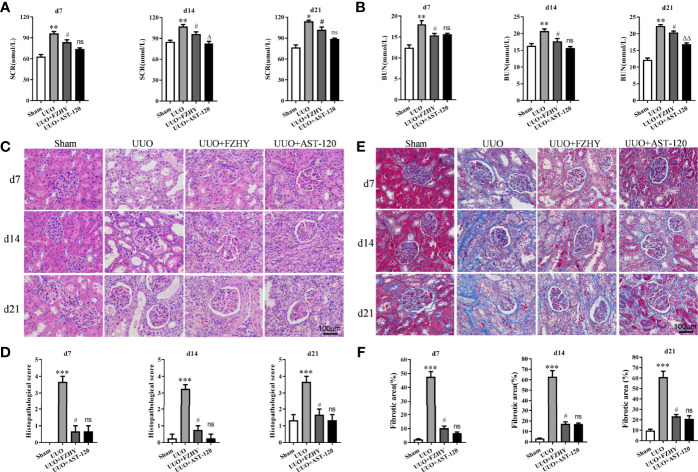
Effect of FuZhengHuaYuJiangZhuTongLuoFang prescription (FZHY) on renal function in unilateral ureteral obstruction (UUO) rats. Levels of the biochemical parameters including serum creatinine (SCR) **(A)** and blood urea nitrogen (BUN) **(B)** were detected in the sham, UUO, UUO+FZHY, and UUO+AST-120 groups on days 7, 14, and 21. The renal tissue injury was detected **(C)** and scored **(D)** based on histology (HE staining). The renal tissue fibrosis was detected **(E)** and the relative fibrotic area was analyzed **(F)** based on Masson’s trichrome staining; ×400 magnification on days 7, 14, and 21. *P < 0.05, **P < 0.01, ***P < 0.001, the UUO group vs. the sham group; ^#^ *P* < 0.05, the UUO+FZHY group vs. the UUO group; ^Δ^ *P* < 0.05, ^ΔΔ^ *P* < 0.01, the UUO+AST-120 group vs. the FZHY-treated group. ns, not significant.

The functional parameters were used to assess whether FZHY improved renal function, and the results showed that FZHY significantly reduced the serum SCR and BUN levels on days 7, 14, and 21 in the UUO model. There were no statistically significant differences between the UUO+FZHY group and the AST-120 group on day 7 and slight differences on days 14 and 21 (**Figures 1A, B** **)**. Meanwhile, the HE staining results showed renal tissue structure damage, renal tubule dilatation, partial renal tubule epithelial cell shedding, and hyperemia on days 7, 14, and 21 in the UUO model compared with the sham group. However, FZHY treatment significantly reversed these results (**Figures 1C, D** **)**. Furthermore, the Masson staining results showed severe renal fibrosis on days 7, 14, and 21 in the UUO model compared with the sham group, whereas fibrosis was also significantly reversed by FZHY treatment (**Figures 1E, F** **)**.

### FZHY Treatment Improved the Colonic Function in UUO Rats

**Figure 2:**
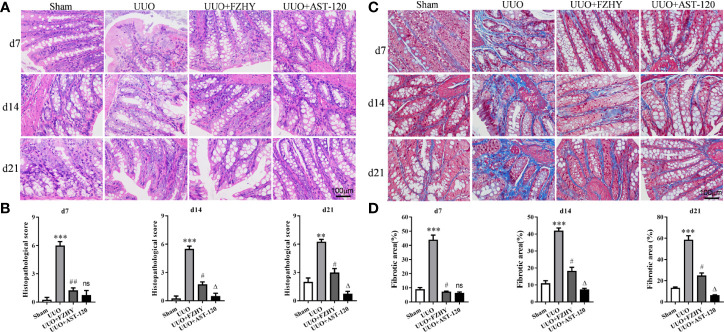
Effect of FZHY on colon tissue in UUO rats. The colon tissue injury was assessed **(A)** and scored **(B)** based on HE staining. The colon tissue fibrosis was assessed **(C)** and scored **(D)** based on Masson’s trichrome staining; ×400 magnification on days 7, 14, and 21. **P < 0.01, ***P < 0.001, the UUO group vs. the sham group; ^#^ *P* < 0.05, ^##^ *P* < 0.01, the UUO+FZHY group vs. the UUO group; ^Δ^ *P* < 0.05, the UUO+AST-120 group vs. the FZHY-treated group. ns, not significant.

CKD is usually accompanied by an impaired colonic mucosal barrier (Ji et al., 2020). The HE staining results showed colon tissue damage in the UUO model, with epithelial cells damaged and goblet cells reduced, whereas FZHY treatment improved these results. Meanwhile, the effect of FZHY on improving intestinal injury was most obvious on day 7 (**Figures 2A, B** **)**. Furthermore, the Masson staining showed obvious colon fibrosis in UUO rats, but it was significantly attenuated by FZHY. Likewise, the improvement was most noticeable on day 7 (**Figures 2C, D** **)**.

### FZHY Treatment Improved Microbiota Imbalance in UUO Rats

**Figure 3:**
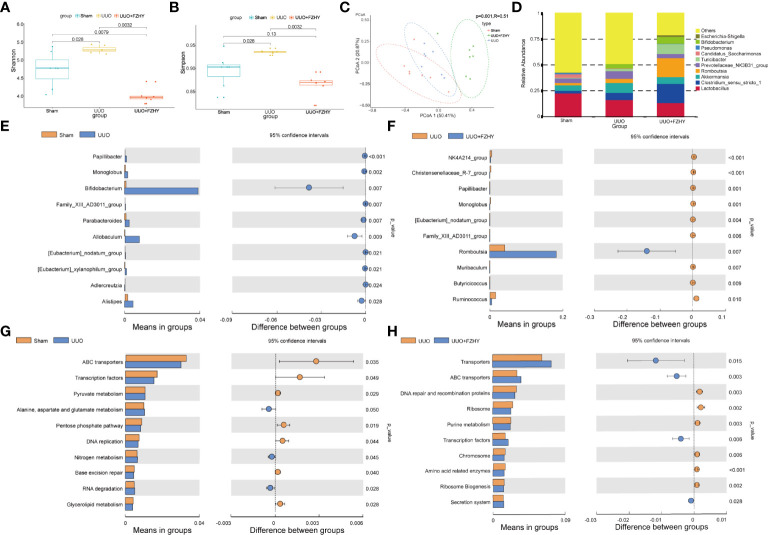
Diversity and abundance analysis. The alpha diversity [Shannon **(A)**, Simpson **(B)**] based on operational taxonomic unit (OTU) abundance for the sham, UUO, and UUO+FZHY groups. **(C)** The beta diversity shows the PCoA plot of UniFrac distances. **(D)** The top 10 high relative abundance for the sham, UUO, and UUO+FZHY groups. The difference of abundance at the genus level in the sham group compared with the UUO group **(E)** and the UUO group compared with the UUO+FZHY group **(F)**. The difference of abundance at the genus level in the sham group compared with the UUO group **(G)** and the UUO group compared with the UUO+FZHY group **(H)**. Wilcoxon rank-sum test was used to compare the difference in diversity between the two groups in the boxplot. *t*-test was used to compare the difference in abundance between the two groups; *P <*0.05 is considered to be a significant difference.

Based on the fact that FZHY significantly improved kidney injury and intestinal injury on the 7th day, 16S sequencing was performed on the intestinal flora of the three groups to observe whether FZHY adjusted the changes of intestinal flora to adapt to environmental changes. The alpha diversity including Shannon and Simpson indices of the gut microbiota among the sham, UUO, and UUO+FZHY groups was compared. The results showed that microbial community diversity was higher in the gut microbiome of UUO rats than that of the sham group, while that of the FZHY treatment group was significantly lower than that of the UUO group (**Figures 3A, B** **)**. The beta diversity of the gut microbiota among the sham, UUO, and FZHY groups was compared, and the corresponding results of the PCoA plots showed a significant difference of flora composition at the genus level among the sham, UUO, and UUO+FZHY groups (**Figure 3C**). Furthermore, at the genus level, the colonic 16S rRNA sequencing data of the sham group, the UUO group, and the FZHY group all belonged to the 10 most common genera: *g_Lactobacillus*, *g_Prevotellaceae_NK3831_group*, *g_Clostridium_sensu_stricto_1*, and other seven genera (**Figure 3D**). Moreover, the *t*-test analysis was used to identify the top 10 most significant taxonomy biomarkers that could differentiate the sham, UUO, and UUO+FZHY groups. The relative abundance of *g_Papillibacter*, *g_Bifidobacterium*, *g_Monoglobus*, *g_Allobaculum*, *g_Family_XIII_AD3011_group*, *g_Parabacteroides*, *g_[Eubacterium]_xylanophilum_group*, *g_Adlercreutzia*, *g_Faecalibaculum*, and *g_[Eubacterium]_nodatum_group* were all enriched in the UUO group than in the sham group (**Figure 3E**), while *g_Monoglobus*, *g_NK4A214_group*, *g_Christensenellaceae_R-7_group*, *g_Papillibacter*, *g_[Eubacterium]_nodatum_group*, *g_Muribaculum*, *g_Romboutsia*, *g_Family_XIII_AD3011_group*, *g_Ruminococcus*, and *g_Turicibacter* were all significantly decreased in the UUO+FZHY group than in the UUO group (**Figure 3F**). Among them, four pathogenic bacteria increased in UUO but decreased in the sham and UUO+FZHY groups. Furthermore, we also selected the top 10 significantly enriched pathways among the sham, UUO, and UUO+FZHY groups. The results showed that pyruvate metabolism; alanine, aspartate, and glutamate metabolism; nitrogen metabolism; and others were enriched in the UUO group relative to the sham group (**Figure 3G**), while purine metabolism, amino acid-related enzymes, and others were decreased in the UUO+FZHY group when compared with the UUO group (**Figure 3H**).

### FZHY Treatment Improved Dysregulation of Plasma Metabolites in UUO Rats

**Figure 4:**
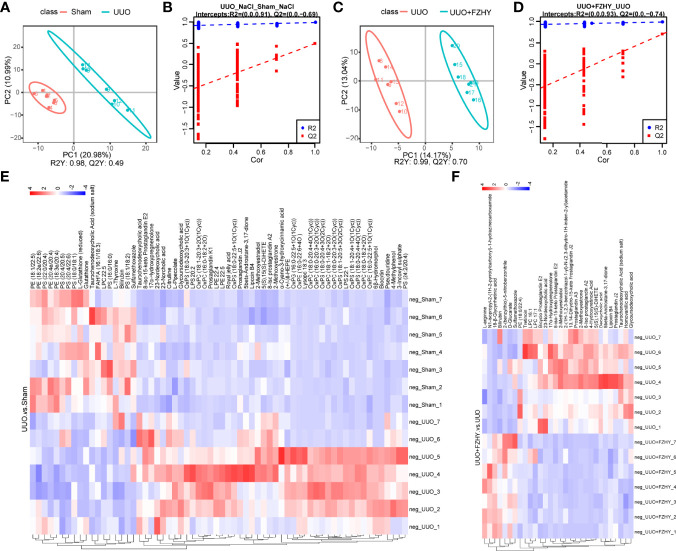
Metabolomics analysis of cationic metabolites. PLS-DA score plots for the comparison of the global metabolite profiles in the UUO and sham groups **(A)** and the UUO+FZHY and UUO groups **(C)**. Validation plot by permutation test for the UUO and sham groups **(B)** and the UUO+FZHY and UUO groups **(D)**; the abscissa represents the correlation between the Y of the random grouping and the original group Y, and the ordinate represents the scores of R2 and Q2. R2Y represents the interpretation rate of the model, Q2Y is used to evaluate the predictive ability of the PLS-DA model, and a higher R2Y value in a PLS-DA model indicates a better model fit. The heatmap plot for metabolites between the UUO vs. sham groups **(E)** and the UUO+FZHY vs. UUO groups **(F)**.

**Figure 5:**
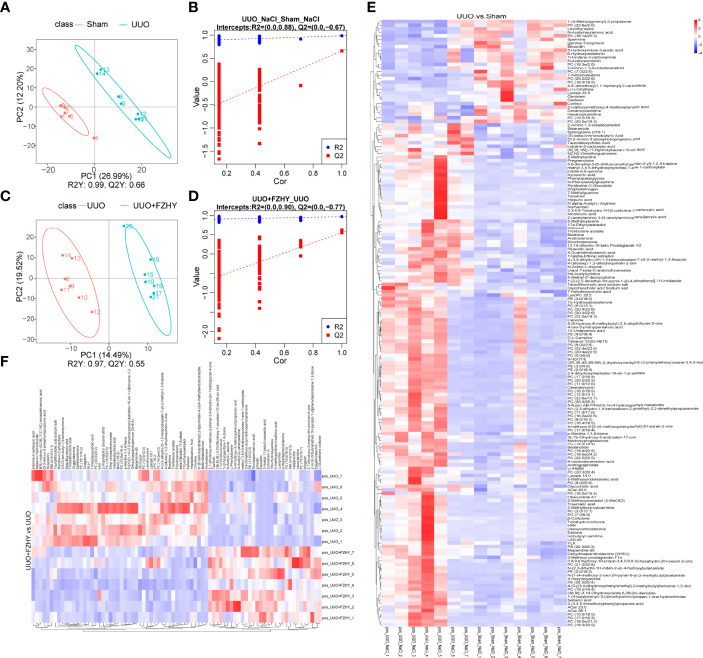
Metabolomics analysis of anionic metabolites. PLS-DA score plots for the comparison of the global metabolite profiles in the UUO and sham groups **(A)** and the UUO+FZHY and UUO groups **(C)**. Validation plot by permutation test for the UUO and sham groups **(B)** and the UUO+FZHY and UUO groups **(D)**. The heatmap plot for metabolites between the UUO vs. sham groups **(E)** and the UUO+FZHY vs. UUO groups **(F)**.

To assess the effects of FZHY on gut microflora imbalance-related metabolic pathways, the untargeted metabolomic analyses of rat plasma on day 7 using the LC-MS technique were performed. The positive and negative ion mode analysis was as follows. The PLS-DA score plots showed a significant difference in plasma metabolites between the sham and UUO groups and the UUO and UUO+FZHY groups (**Figures 4A, C**, **5A, C**). The PLS-DA valid results showed that the model is stable and accurate (**Figures 4B, D**, **5B, D**). Furthermore, the heatmap results showed 46 upregulated and 18 downregulated metabolites in the UUO group compared with the sham group (**Figure 4E**) and 7 upregulated and 23 downregulated metabolites in the UUO+FZHY group compared with the UUO group in negative ion (**Figure 4F**). Meanwhile, there were 142 upregulated and 29 downregulated metabolites in the UUO group compared with the sham group (**Figure 5E**) and 31 upregulated and 48 downregulated metabolites in the UUO+FZHY group compared with the UUO group in positive ion (**Figure 5F**). The heatmap plot for both the positive and negative ion metabolites showed that samples can be well divided into two groups, separately. Significant differences of metabolites were identified belonging to amino acids, lipids, organic acids, bile acids, uremic toxins, hormones, etc., such as l-pipecolate, citrulline, PS (18:1/18:2), PS (20:3/20:3), N4-acetylcytidine, taurochenodeoxycholic acid, deoxycholic acid, nordeoxycholic acid, ursodeoxycholic acid, 4-methylpheno, 3-indoxyl sulfate, phenylacetylglycine, iso prostaglandin A2, and trenbolone acetate. Among them, 36 metabolites with significant differences were simultaneously identified in the sham, UUO, and UUO+FZHY groups.

### Relationship Between Microbial Dysregulation and Metabolic Product Dysregulation

**Figure 6:**
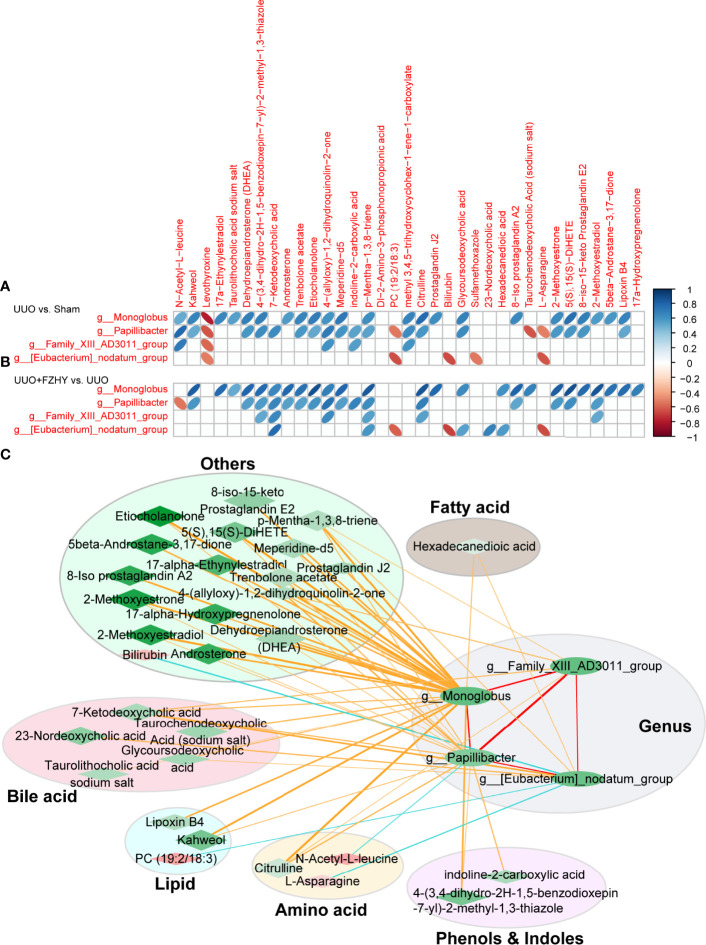
Correlation analysis between differential genus and differential metabolites. The correlation heatmaps for the UUO vs. sham groups **(A)** and the UUO+FZHY vs. UUO groups **(B)** were analyzed by Pearson correlation coefficient. The “blue” bar represents positive correlation, and the “orange” bar represents negative correlation. **(C)** The correlation network between 4 genus-level bacterial taxa and 31 metabolites by the Cytoscape software. The nodes of the network represent the genera (green ellipse) and the metabolites (red diamond and green diamond). “Red” represents upregulation and “green” represents downregulation. The node color represents the level of difference (logFC) between the two groups (FZHY vs. UUO), and the darker the color, the greater the difference. The diamond size represents the −log10 (*P*_value) of correlation; the larger of the significance, the higher of the correlation, and vise versa. The edges represent the degree of correlation between the two nodes. The color of the edge represents the correlation between the two; “yellow” and “red” represent positive correlation and “blue” represents negative correlation. The thickness of the edge represents the degree of correlation, and the thicker the edge, the higher the correlation. The metabolites are divided into six categories, namely, bile acid metabolites (light pink ellipse), lipid metabolites (light blue ellipse), amino acid metabolites (light yellow ellipse), phenol and indole metabolites (light purple ellipse), fatty acid metabolites (light gray ellipse), and other metabolites (light green ellipse).

Intestinal flora is involved in the metabolism of abnormal metabolites during the progression of CKD. Therefore, we used Pearson correlation analysis to analyze the correlation between the above four potential bacterial biomarkers and 35 potential plasma metabolite markers screened from these three groups, so as to explore the metabolism of potential bacterial biomarkers involved in the early stage of CKD. As expected, 27 and 5 differential metabolites were decreased and increased in the sham and FZHY groups compared with the UUO group and were positively and negatively associated with the four potential bacteria biomarkers, namely, *g_Monoglobus*, *g_Papillibacter*, *g_Family_XIII_AD3011_group*, and *g_Eubacterium_nodatum_group* in the FZHY group, respectively. Between the sham and UUO groups, 25 and 6 differential metabolites were increased and decreased and were positively and negatively associated with the four potential bacteria biomarkers in UUO rats (**Figure 6A**). Between the UUO and UUO+FZHY groups, 27 and 4 differential metabolites were decreased and increased and were positively and negatively associated with the four potential bacteria biomarkers in UUO+FZHY rats (**Figure 6B**). Furthermore, to explore the association between bacterial genus and metabolites, a network analysis based on 4 genus-level bacterial taxa and 31 metabolites was used to highlight the associations of the gut microbiome and plasma metabolites in FZHY-treated UUO rats by the Cytoscape software. The results reveal that alterations of the metabolites were mainly involved in bile acid metabolism, lipid metabolism, amino acid metabolism, phenol and indole metabolism, fatty acid metabolism, and others in FZHY-treated UUO rats and associated with four candidate bacteria genera (**Figure 6C**).

### FZHY Treatment Reduced the Colonic Epithelial Barrier Damages of UUO Rats

**Figure 7:**
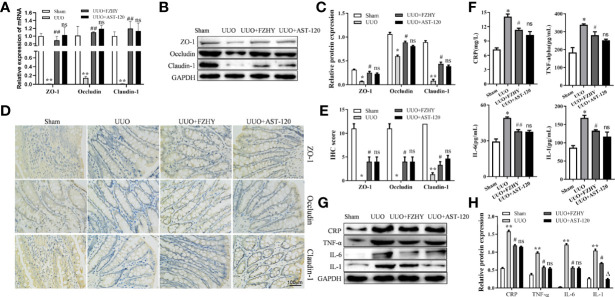
Effect of FZHY on the integrity of intestinal tissue barrier in UUO rats. The mRNA **(A)** and protein **(B, C)** levels of the colon tissue tight junction proteins ZO-1, occludin, and claudin-1 were evaluated in the sham, UUO, UUO+FZHY, and UUO+AST-120 groups on day 7. The immunohistochemistry of ZO-1, occludin, and claudin-1 from kidney tissue sections was analyzed **(D, E)**. The secretion levels of the inflammation molecules including CRP, IL-1, IL-6, and TNF-α were measured in the sham, UUO, UUO+FZHY, and UUO+AST-120 groups on day 7 **(F)**. The protein levels **(G, H)** of the serum inflammation molecules in the sham, UUO, UUO+FZHY, and UUO+AST-120 groups on day 7. *P < 0.05, **P < 0.01, the UUO group vs. the sham group; ^#^ *P* < 0.05, ^##^ *P* < 0.01, the UUO+FZHY group vs. the UUO group; ^Δ^ *P* < 0.05, the UUO+AST-120 group vs. the FZHY-treated group. ns, not significant.

CKD causes damage to the intestinal barrier, leading to serum cytokine production and kidney damage (Lau et al., 2018). As expected, at the early stage of CKD, the mRNA and protein expression levels of intestinal epithelial tight junction molecules such as ZO-1, occludin, and claudin-1 were decreased in the UUO group compared with the sham group, while FZHY treatment obviously reserved their expression levels (**Figures 7A–C**). Furthermore, the IHC results also showed that the levels of ZO-1, occludin, and claudin-1 were obviously reduced in the colonic mucosa of the UUO model, while FZHY reversed their high levels in the UUO groups (**Figures 7D, E** **)**. In addition, the secretion levels of inflammatory cytokines including CRP, TNF-α, IL-6, and IL-1 were increased in the UUO serum (**Figure 7F**). On the contrary, the UUO+FZHY group protects the kidney by reducing the expression levels of CRP, TNF-α, IL-6, and IL-1 in the UUO model (**Figures 7G, H** **)**.

### FZHY Treatment Reduced Renal Tissue Fibrosis of UUO Rats

**Figure 8:**
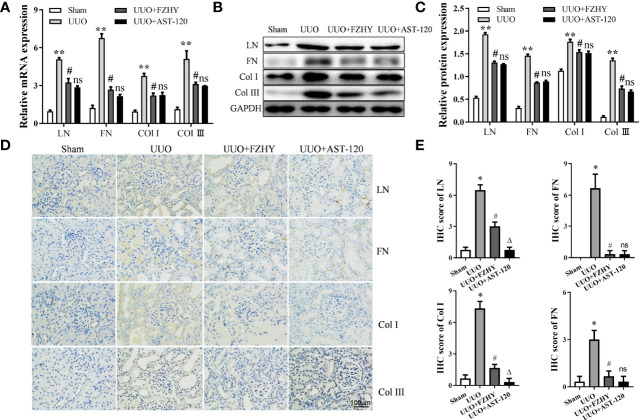
Effect of FZHY on renal interstitial fibrosis in UUO rats. The mRNA **(A)** and protein **(B, C)** levels of the renal interstitial fibrosis proteins LN, FN, Col-I, and Col-III were evaluated in the sham, UUO, UUO+FZHY, and UUO+AST-120 groups on day 7. The immunohistochemistry of LN, FN, Col-I, and Col-III **(D, E)** from kidney sections was analyzed. *P < 0.05, **P < 0.01, the UUO group vs. the sham group; ^#^ *P* < 0.05, the UUO+FZHY group vs. the UUO group. ns, not significant.

Compared with the sham group, the mRNA and protein expression levels of the renal fibrosis-related markers LN, FN, Col-I, and Col-III were increased on day 7 in the renal tissues of the UUO group. However, their expression levels were reversed in the UUO+FZHY group (**Figures 8A–C**). In addition, the results of IHC also showed that UUO treatment effectively increased the deposition of LN, FN, Col-I, and Col-III in the tubulointerstitium of the rats and also showed obvious renal damage such as tubular dilation. However, FZHY treatment markedly blunted their deposition (**Figures 8D, E** **)**.

## Discussion

**Figure 9:**
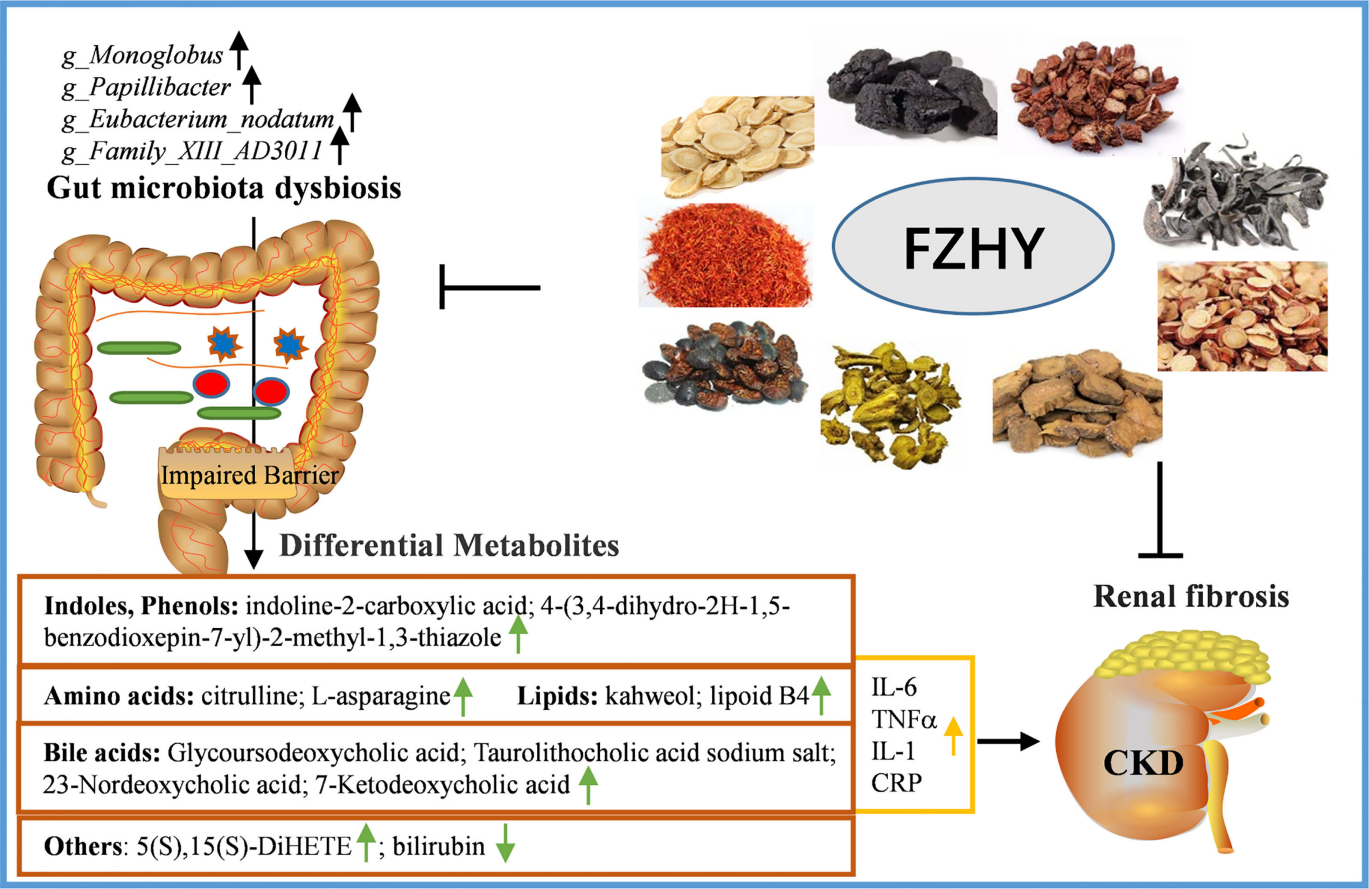
A schematic diagram of the mechanisms of FZHY protecting the pathogenesis of CKD by regulating intestinal flora and its related metabolites.

Disturbance in the composition of the gut microbiota mediates a series of adverse reactions in host metabolism, thereby affecting the process and development of CKD. However, a large number of studies on the gut microbiome of CKD have focused on the advanced stages of the disease (Vaziri et al., 2013a; Crespo-Salgado et al., 2016; Jiang et al., 2017). Therefore, it is very necessary to explore the early CKD microbiome. In our current study, the findings indicate that FZHY at the dose of 4.92 g/kg/day is more effective in treating UUO-induced CKD on day 7, manifested by increased renal function and decreased renal damage in UUO rats. Furthermore, FZHY obviously regulates pathogenic bacteria disorders, reduces intestinal tissue epithelial injury and downstream inflammation, and improves serum metabolite abnormalities. In short, the traditional Chinese medicine FZHY alleviates kidney injury in CKD rats with such a mechanism through the interrelationship between the intestinal flora and serum metabolites (**Figure 9**).

FZHY, an herb medicine containing astragalus, *R. glutinosa*, red sage, safflower, rhubarb, and others (Zhang et al., 2014; Tao et al., 2015; Qin et al., 2020), was widely used in the clinical treatment of CKD in China. This prescription makes up for the deficiency of Shenkang’s blood-activating function and the nourishing effect of the Dahuangzhechong pill. The present study showed that there are significant differences in the α-diversity and β-diversity indices of the intestinal flora of rats in the sham, UUO, and UUO+FZHY groups, indicating the variation in the composition of the intestinal flora among these three groups, which is similar to the results of previous studies (Feng et al., 2019; Wu et al., 2020). As expected, 13 and 16 genera-level differential bacterial biomarkers were identified between sham and UUO, UUO, and UUO+FZHY, respectively. Importantly, among the differentially abundant microbes, it is worth noting that UUO rats treated with FZHY showed that the four genera *g_Monoglobus*, *g_Papillibacter*, *g_Eubacterium_nodatum*, and *g_Family_XIII_AD3011* were all reduced to levels similar with those of sham-operated rats. These results implicate that these specific gut microorganisms may be the candidate biomarkers for early diagnosis and prognosis monitoring of CKD. However, there is currently no research on the direct correlation between these four genera and CKD. A previous study showed that the abundance of *g_Monoglobus* in the hindgut of pigs is positively correlated with aerial ammonia concentration (Tang et al., 2021). Previous studies have stated that respiratory ammonia is an important marker for patients with renal failure and is directly related to blood ammonia level caused by decreased excretion in patients with CKD (Narasimhan et al., 2001; Jankowski et al., 2014). It was reported that *g_Monoglobus* has been proven to be an efficient pectin-degrading bacteria in the human colon (Kim et al., 2019), and the reduction of fermentable fiber can increase the amount of blood ammonia absorbed by the portal system (Amodio et al., 2013). Therefore, we speculate that *g_Monoglobus* is positively correlated with blood ammonia levels. Elevated ammonia levels have been shown to promote the destruction of the intestinal epithelial barrier, leading to the inflammatory pathogenesis of CKD (Vaziri et al., 2013b). These studies also confirmed our results that intestinal barrier disruption in UUO rats was characterized by decreased ZO-1, occludin, and claudin-1 and increased inflammation mainly by increased secretion of CRP, TNF-α, IL-6, and IL-1. However, FZHY treatment restored the decrease of these tight junction markers in intestinal epithelial tissue and the secretion of these inflammatory factors, suggesting that it participated in intestinal protection and inflammation resistance, which were also similar to previous studies (Pahl and Vaziri, 2015; Asai et al., 2019). Intestinal barrier damage is associated with intestinal microbiota disturbance (Ji et al., 2020). These studies confirmed our results, that is, *g_Monoglobus* dysregulation regulated by FZHY may be related to the destruction of the intestinal barrier in UUO rats and the increase of the main pro-inflammatory factors in UUO rat serum. Increased inflammation is a typical feature of early CKD and is one of the pathological mechanisms leading to sustained renal impairment (Lukaszyk et al., 2018). In our previous studies, FZHY was shown to ameliorate chronic renal failure by ameliorating the “evil excess” condition caused by microinflammation in five-sixths of the nephrectomy model (Chen et al., 2021). In addition, a previous study showed that the increased relative abundance of *g_Papillibacter* is higher in primary IgA nephropathy than that in controls (De Angelis et al., 2014), which is similar to our research results. In addition, the enrichment of *g*_*Family_XIII_AD3011_group* is present in the colon and has been reported to be closely associated with indole production (Shi et al., 2020), the most common toxin in CKD patients or animal models. The pathogenic bacteria *Eubacterium_nodatum_group* disorder has been found in cerebral ischemic stroke patients with the main complications of dyslipidemia and increased blood viscosity (Chen et al., 2019). Taken together, the synergistic effect of these potential bacteria biomarkers may be the decisive factor for FZHY to improve the pathogenesis of early CKD.

The imbalance of intestinal flora in CKD rats is accompanied by changes in serum metabolites. As expected, significant differences were identified in 219 plasma metabolites involved in lipid, amino acid, and bile acid metabolism in the sham, UUO, and UUO+FZHY groups. Importantly, alteration of the same 36 metabolisms in our UUO model and the FZHY group was associated with four potential bacterial biomarkers. Among them, the enrichment of *g_Monoglobus* in UUO rats was positively associated with citrulline. Citrulline is a non-essential amino acid, a protective agent to prevent ammonia toxicity, and completes the detoxification function by stimulating the formation of urea (Summar and Mew, 2018). Increased citrulline in plasma has been reported in humans and animals with impaired renal function, which may be due to reduced renal uptake capacity (Ceballos et al., 1990). Studies have shown that plasma citrulline levels are relatively elevated in patients with initial CKD (Chen and Baylis, 2010; Duranton et al., 2014). Our research also showed that citrulline in UUO decreased significantly after FZHY treatment, which was significantly negatively correlated with *g_Monoglobus*. Therefore, we speculate that FZHY reduces the relative abundance of intestinal *g_Monoglobus* in UUO rats, thereby reducing blood ammonia and citrulline levels, contributing to improving renal damage, and weakening the development of CKD. In addition, *g_Monoglobus* is positively correlated with kahweol, taurolithocholic acid sodium salt, 4-(3,4-dihydro-2H-1,5-benzodioxepin-7-yl)-2-methyl-1,3-thiazole, p-mentha-1,3,8-triene, glycoursodeoxycholic acid, 5(S),15(S)-DiHETE, lipoxin B4, and several alcohol hormones. Kahweol is a lipid component that is positively correlated with the increased concentration of cholesterol in human plasma (Ranheim and Halvorsen, 2005), and an abnormal increase of cholesterol is also an abnormal metabolic manifestation of CKD. Bile acid is widely accumulated in the serum of CKD patients and animals (Wang et al., 2020). The taurolithocholic acid sodium salt that we identified is a type of bile acid, and its increase may aggravate CKD disease. P-cresyl sulfate is a uremic toxin derived from intestinal microbes (Koppe et al., 2013). 4-(3,4-Dihydro-2H-1,5-benzodioxepin-7-yl)-2-methyl-1,3-thiazole is the precursor of p-cresyl sulfate and may be closely related to the progression of CKD (Meijers and Evenepoel, 2011). Glycoursodeoxycholic acid is a novel uremic solute in the kidney and its long-term accumulation is typical of CKD (Boelaert et al., 2017). In addition, 5(S),15(S)-DiHETE and lipoxins are inflammation markers derived from leukocytes (Borgeat et al., 1982; Chavis et al., 1995), and their increase in UUO rats also resulted in an increase in pro-inflammatory activity. In all, these results confirmed our findings of plasma metabolites in UUO rats, and the restoring effect of FZHY also implies that FZHY has the ability to improve the composition of these metabolites in CKD. In addition, indoline-2-carboxylic acid is an indole-derivative toxic metabolite, and like other indoles, its concentration increases in the plasma of patients with renal failure (Bowmer and Lindup, 1982). The correlation analysis results showed that *g_Papillibacter* and *g_Family_XIII_AD3011_group* also showed similar correlations mainly positively correlated with the obvious reduction of 7-ketodeoxycholic acid, indoline-2-carboxylic acid, and citrulline levels in rats in the FZHY group, suggesting that *g_Papillibacter* and *g_Family_XIII_AD3011_group* may increase the levels of bile acid and toxins and promote inflammation in UUO rats. This may be similar to the previous research mentioned above (De Angelis et al., 2014; Shi et al., 2020). Furthermore, the decrease in the abundance of *Eubacterium_nodatum_group* in FZHY is significantly positively correlated with 23-nordeoxycholic acid but inversely associated with bilirubin and L-asparagine. 23-Nordeoxycholic acid is also a bile acid that may be involved in the progression of CKD. In addition, it was reported that low levels of bilirubin have been found in peritoneal dialysis patients who have died (Fan, 2019). Taken together, the regulation of intestinal flora composition is the target to alleviate the progress of CKD. It has been reported that AST-120 regulates intestinal flora such as *Lactobacillus* and *Escherichia coli* in the intestinal environment, affecting toxins and inflammation produced by gut microbiota and ultimately delaying the decline of renal function (Yoshifuji et al., 2018; Hirakawa et al., 2020). At present, FZHY treatment ameliorates the imbalance of these candidate bacterial biomarkers in CKD; reduces the production of harmful metabolites such as serum ammonia, toxin, cholic acid, and inflammation; and alleviates the tubulointerstitial fibrosis.

In summary, the present study implicates that the use of the Chinese herb FZHY effectively modulates intestinal flora disorders in UUO rats and inhibits gut-derived harmful metabolites, which may thus be a mechanism related to the beneficial effect of FZHY on CKD. These findings suggest that FZHY may serve as a potential therapeutic agent and the four candidate bacteria are expected to be therapeutic targets in the future.

## Data Availability Statement

The datasets presented in this study can be found in online repositories. The names of the repository/repositories and accession number(s) can be found below: CNGBdb (https://db.cngb.org/cnsa/) (Guo et al., 2020; Chen et al., 2020) CNP0002488; CNP0002493.

## Ethics Statement

The animal study was reviewed and approved by the Ethics Committee of Chengdu University of Traditional Chinese Medicine.

## Author Contributions

MC contributed to the study conception and design. Material preparation and data collection and analysis were performed by ZWC and SBW. The first draft of the manuscript was written by ZWC. YZ, ZJC, XYL, JL, and LH were responsible for data visualization and literature search. MC was a major contributor in critically revising the manuscript. All authors commented on previous versions of the manuscript and read and approved the final manuscript.

## Funding

This study was supported by the National Natural Science Foundation of China (No. 81973673 to MC) and the Science and Technology Foundation of Sichuan Province (No. 2021YFS0034 to MC).

## Conflict of Interest

The authors declare that the research was conducted in the absence of any commercial or financial relationships that could be construed as a potential conflict of interest.

## Publisher’s Note

All claims expressed in this article are solely those of the authors and do not necessarily represent those of their affiliated organizations, or those of the publisher, the editors and the reviewers. Any product that may be evaluated in this article, or claim that may be made by its manufacturer, is not guaranteed or endorsed by the publisher.
